# Structure of 2,3,5-tri­phenyl­tetra­zol-3-ium chloride hemi­penta­hydrate

**DOI:** 10.1107/S205698902400940X

**Published:** 2024-09-30

**Authors:** Rao M Uppu, Krishnaveni Chikkula, Soheil Saneei, Sainath Babu, Frank R. Fronczek

**Affiliations:** a Department of Environmental Toxicology Southern University and A&M College Baton Rouge LA 70813 USA; b Department of Chemistry Louisiana State University,Baton Rouge LA 70803 USA; University of Aberdeen, United Kingdom

**Keywords:** crystal structure, redox indicator, cellular respiration, tetra­zolium reduction assays

## Abstract

The title hydrated mol­ecular salt, C_19_H_15_N_4_^+^·Cl^−^·2.5H_2_O, has two tri­phenyl­tetra­zolium cations, two chloride anions and five water mol­ecules in the asymmetric unit. The cations differ in the conformations of the phenyl rings with respect to the heterocyclic core, most notably for the C-bonded phenyl ring, for which the N—C—C—C torsion angles differ by 36.4 (3)°. This is likely a result of one cation accepting an O—H⋯N hydrogen bond from a water mol­ecule [O⋯N = 3.1605 (15) Å], while the other cation accepts no hydrogen bonds. In the extended structure, the water mol­ecules are involved in centrosymmetric (H_2_O)_2_Cl_2_ rings as well as (H_2_O)_4_ chains. An unusual O—H⋯π inter­action and weak C—H⋯O and C—H⋯Cl hydrogen bonds are also observed.

## Abstract 2

The packing of the title hydrated mol­ecular salt features an unusual O—H⋯π inter­action.

## 1. Chemical context

2,3,5-Tri­phenyl­tetra­zolium chloride, commonly known as tetra­zolium red or TTC, is a versatile redox indicator extensively used in biochemical experiments, especially for evaluating cellular viability (Rich *et al.*, 2001 ▸) and seed quality control in various crops (França-Neto & Krzyzanowski, 2019 ▸). Beyond these applications, TTC demonstrates inducible antagonistic activity in the Bacillales effective against a host of microbes including *R. solanacearum*, *E. coli*, and *Staphylococcus sp* (Sierra-Zapata *et al.*, 2020 ▸). Furthermore, the utility of TTC extends to infarct (localized dead tissue) measurement of the brain and heart in experimental animal studies (Sanchez-Bezanilla *et al.*, 2021 ▸), validation of automated colony counting systems (Frost *et al.*, 2016 ▸), and studying heat tolerance in cotton (Jaconis *et al.*, 2021 ▸), as well as assessing fine-root vitality in coniferous forest stands (Clemensson-Lindell, 1994 ▸). Despite its widespread use, the crystallographic aspects of TTC have been relatively unexplored, and we now describe the crystal structure of the title hydrated mol­ecular salt, C_19_H_15_N_4_^+^ Cl^−^·2.5H_2_O (I),

This is important, because the nuances in the conformations of the pendant tetra­zolium rings may influence the transport mechanisms of TTC across biological membranes and its reduction by mitochondrial NADH: ubi­quinone oxidoreduc­tase (Complex 1) or other cellular sites (Ling *et al.*, 1957 ▸; Rich *et al.*, 2001 ▸). Exploring these structural intricacies is expected to deepen our comprehension of the various applications of TTC, ranging from assessing cell viability to seed testing, measuring infarcts, and exploring its anti­microbial quorum-sensing properties.
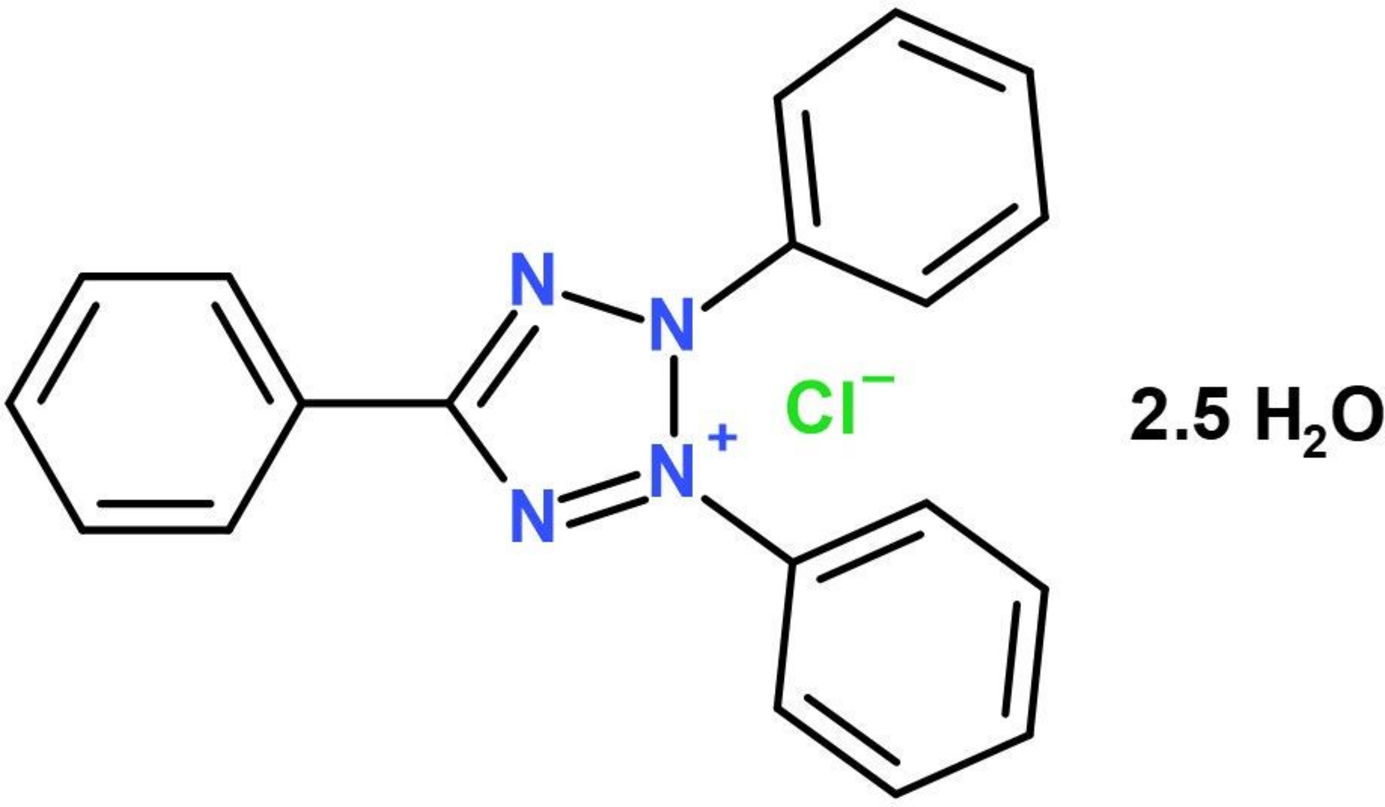


## 2. Structural commentary

**Figure 1:**
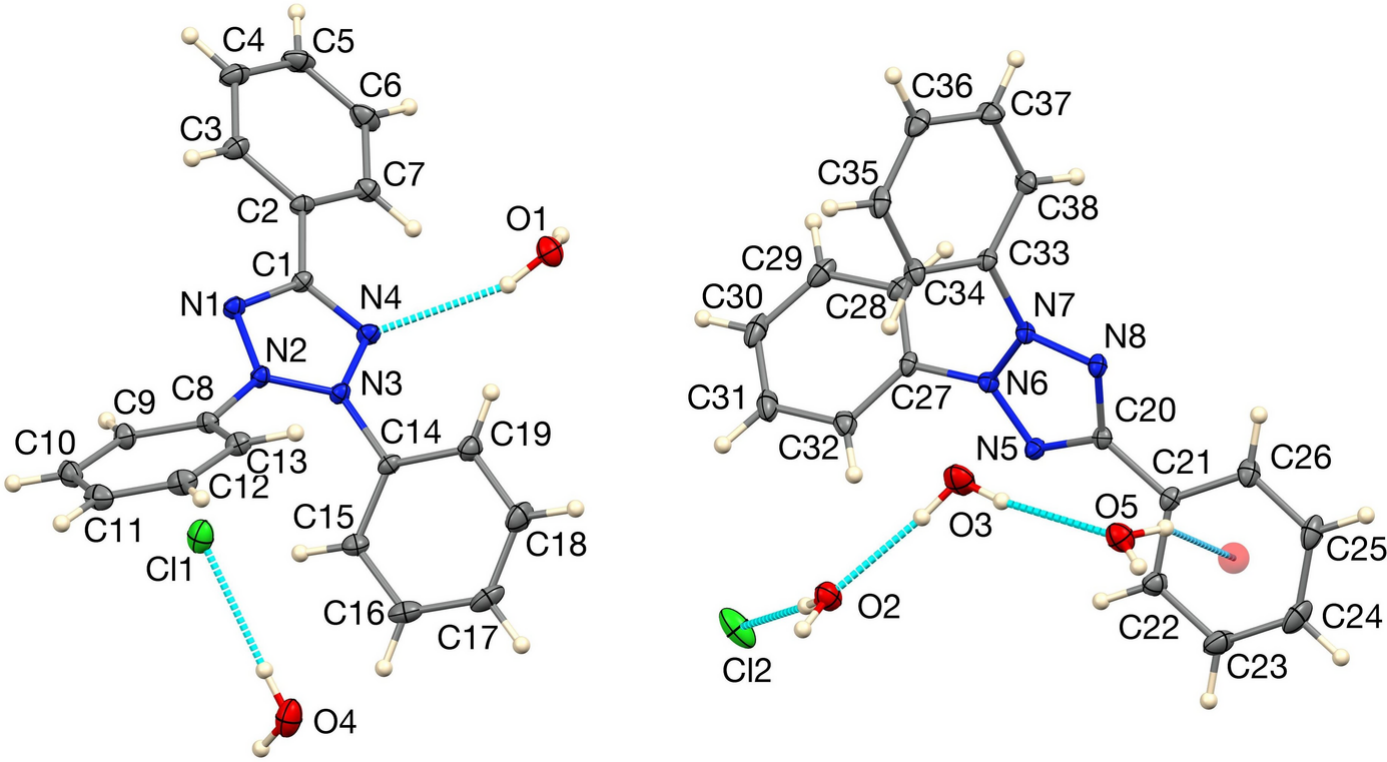
The asymmetric unit of (I) showing 50% displacement ellipsoids. Hydrogen bonds are indicated by dashed lines and the orange circle represents the centroid of the C21–C26 ring.

**Figure 2:**
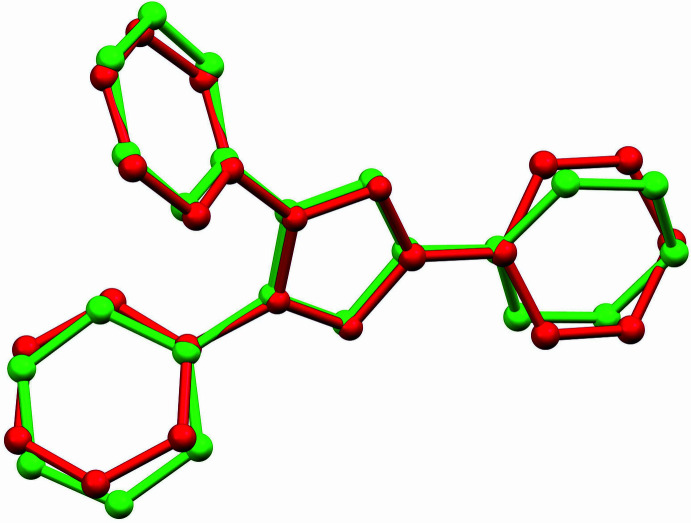
Superimposed structures of the N1 and N5 tri­phenyl­tetra­zolium cations in (I).

The asymmetric unit of (I) is shown in Fig. 1 ▸. The central N—N distance in the heterocycle is 1.3341 (14) Å in the N1 mol­ecule and 1.3324 (14) Å in the N5 mol­ecule, while the other heterocyclic N—N distances are in the range 1.3066 (15) to 1.3137 (15) Å. The heterocyclic C—N distances are in the range 1.3442 (16) to 1.3500 (16) Å over the two cations. The two independent tetra­zolium cations differ somewhat in their phenyl group conformations, one having N—N—C—C torsion angles about the N—C(phen­yl) bond of 48.25 (18)° for N3—N2—C8—C13 and 50.30 (18)° for N2—N3—C14—C15 and the other having corresponding torsion angles of 57.24 (17)° for N7—N6—C27—C28 and 61.37 (17)° for N6—N7—C33—C34. The C-bound phenyl group also differs in conformation, having an N1—C1—C2—C3 torsion angle of 12.26 (19)° in one cation and N5—C20—C21—C22 = −24.14 (19)° in the other. These conformational differences are apparent in the overlay plot, Fig. 2 ▸ (Macrae *et al.*, 2020 ▸). They may result from the fact that the N1 cation accepts a hydrogen bond from a water mol­ecule with an O⋯N distance of 3.1605 (15) Å (Table 1 ▸), while the other does not.

## 3. Supra­molecular features

**Figure 3:**
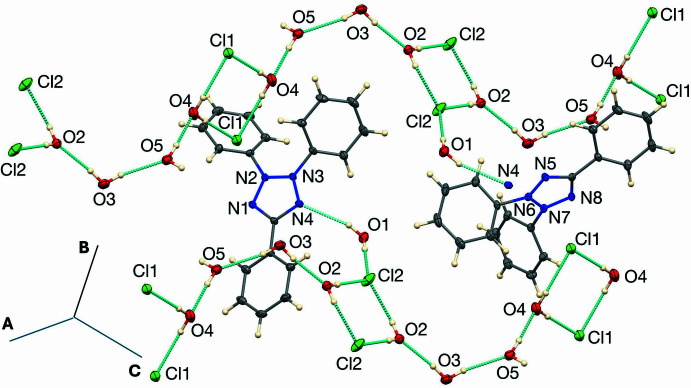
Hydrogen bonding (dashed blue lines) in (I).

**Figure 4:**
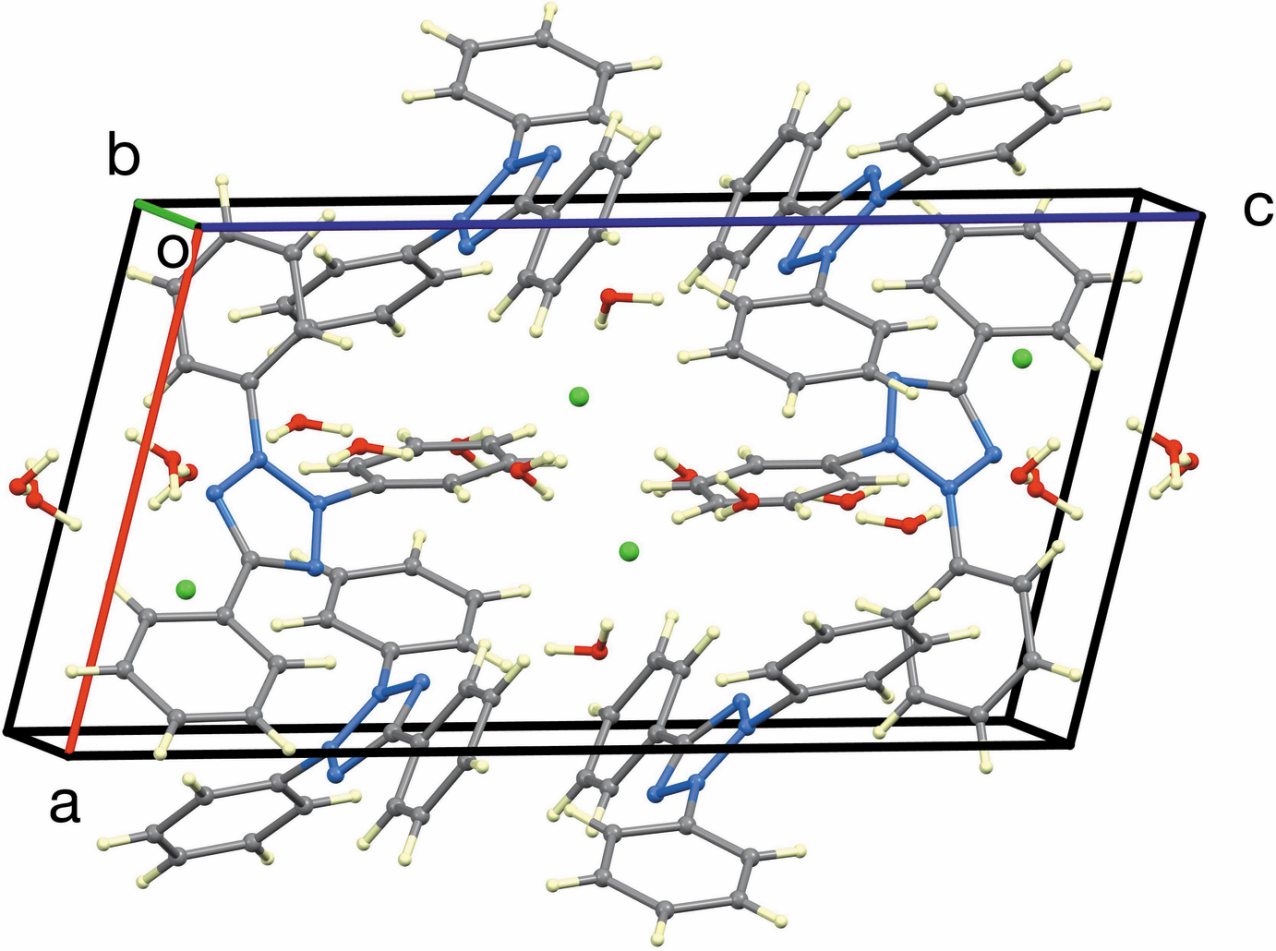
View of the unit-cell packing of (I).

The hydrogen bonding is illustrated in Fig. 3 ▸ and the unit-cell packing is shown in Fig. 4 ▸. As mentioned in the *Structural commentary*, one of the two independent cations accepts no hydrogen bonds, while the other accepts an O—H⋯N hydrogen bond from a water mol­ecule. The remaining water mol­ecules and chloride ions form arrays with chains propagating in the [011] direction, consisting of four hydrogen-bonded water mol­ecules, linked by two independent centrosymmetric (H_2_O)_2_Cl_2_ rings having graph-set notation (Etter *et al.*, 1990 ▸) 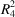
(8). The O4⋯Cl1 distances in one ring are 3.1687 (14) and 3.12008 (14) Å and the O2⋯Cl2 distances in the other ring are 3.1302 (12) and 3.1740 (12) Å. The O⋯O distances in the four-water mol­ecule chain are in the range 2.7503 (17) to 3.0040 (17) Å. The water mol­ecule (O1) that donates a hydrogen bond to a tetra­zolium N atom also donates one to an (H_2_O)_2_Cl_2_ ring, with an O1⋯Cl2 distance of 3.1483 (12) Å. Atom O5 forms an unusual O—H⋯π bond to the C21–C26 benzene ring and various weak C—H⋯O and C—H⋯Cl hydrogen bonds are also observed (Table 1 ▸). The O—H⋯π contact (Allen *et al.*, 1996 ▸; Di Mino *et al.*, 2023 ▸) involves the only water hydrogen atom (H10*W*) that does not donate a conventional hydrogen bond. The H⋯*Cg* distance is 2.76 (2) Å, the O⋯*Cg* distance is 3.4646 (14) Å, and the angle about H is 140.8 (18)°.

## 4. Database survey

We deposited the structure of (I) to the Cambridge Structural Database (CSD, version 5.45, Update 1, March 2024, Groom *et al.*, 2016 ▸) recently as refcode ROJSUI (Chikkula *et al.*, 2023 ▸). A search of the CSD for other salts of the same cation revealed that the structures of 2,3,5-tri­phenyl­tetra­zolium chloride as the aceto­nitrile solvate (LAWXUD), ethanol solvate (LAWYEO) and monohydrate (LAWYAK) have been reported by Golovanov *et al.* (2005 ▸). In addition, the bromide salt ethanol solvate (LEGNUI; Fun *et al.*, 2012*a* ▸) and iodide salt (QECKEQ; Fun *et al.*, 2012*b* ▸) have been described. These structures have a wide range of N—N—C—C torsion angle magnitudes to the N-bound phenyl groups (41.3–86.5°), but a much smaller range of N—C—C—C torsion angle magnitudes to the C-bound phenyl group (2.2–12.6°).

## 5. Synthesis and crystallization

TTC was obtained from Sigma-Aldrich (CAS 298-96-4; purity >98% by HPLC) and was used without purification. Single crystals were prepared by slow cooling of a nearly saturated solution of TTC in boiling distilled water (resistance: 18.2 MΩ cm^−1^).

## 6. Refinement

Crystal data, data collection and structure refinement details are summarized in Table 2 ▸. All H atoms were located in difference maps, and those on C were treated as riding in geometrically idealized positions having C—H = 0.95 Å and *U*_iso_(H) = 1.2*U*_eq_ of the parent C atom. Coordinates of water H atoms were refined with all O—H distances restrained to be approximately equal. Their *U*_iso_ values were set to 1.5*U*_eq_ times their attached O atom.

## Supplementary Material

Crystal structure: contains datablock(s) I, global. DOI: 10.1107/S205698902400940X/hb8107sup1.cif

Supporting information file. DOI: 10.1107/S205698902400940X/hb8107Isup3.cml

Structure factors: contains datablock(s) I Structure factors: contains datablock(s) I. DOI: 10.1107/S205698902400940X/hb8107Isup3.hkl

Structure factors: contains datablock(s) I Structure factors: contains datablock(s) I. DOI: 10.1107/S205698902400940X/hb8107Isup3.hkl

CCDC reference: 2314756

Additional supporting information:  crystallographic information; 3D view; checkCIF report

## Figures and Tables

**Table 1:** Hydrogen-bond geometry (Å, °) *Cg*6 is the centroid of the C21–C26 ring.

*D*—H⋯*A*	*D*—H	H⋯*A*	*D*⋯*A*	*D*—H⋯*A*
O1—H1*W*⋯Cl2^i^	0.85 (1)	2.30 (1)	3.1483 (12)	178 (2)
O1—H2*W*⋯N4	0.85 (1)	2.34 (2)	3.1605 (15)	160 (2)
O2—H3*W*⋯Cl2	0.84 (1)	2.30 (1)	3.1302 (12)	176 (2)
O2—H4*W*⋯Cl2^ii^	0.84 (1)	2.34 (1)	3.1740 (12)	175 (2)
O3—H5*W*⋯O2	0.85 (1)	1.98 (1)	2.8246 (16)	175 (2)
O3—H6*W*⋯O5	0.85 (1)	2.16 (2)	3.0040 (17)	171 (2)
O4—H7*W*⋯Cl1^iii^	0.85 (1)	2.32 (2)	3.1687 (14)	179 (3)
O4—H8*W*⋯Cl1	0.85 (1)	2.35 (2)	3.2008 (14)	178 (2)
O5—H9*W*⋯O4^ii^	0.86 (1)	1.90 (1)	2.7503 (17)	175 (2)
O5—H10*W*⋯*Cg*6	0.85 (1)	2.76 (2)	3.4646 (14)	141 (2)
C4—H4⋯O2^iv^	0.95	2.55	3.461 (2)	160
C11—H11⋯O5^v^	0.95	2.59	3.429 (2)	147
C15—H15⋯Cl1	0.95	2.82	3.6219 (14)	143
C16—H16⋯O5^ii^	0.95	2.48	3.4084 (19)	165
C17—H17⋯O2^ii^	0.95	2.42	3.359 (2)	168
C26—H26⋯O5^vi^	0.95	2.50	3.4440 (19)	173

Symmetry codes: (i) 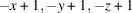; (ii) 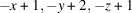; (iii) 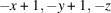; (iv) 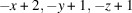; (v) 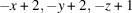; (vi) 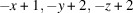.

**Table 2:** Experimental details

Crystal data	
Chemical formula	C_19_H_15_N_4_^+^·Cl^−^·2.5H_2_O
*M*_r_	379.84
Crystal system, space group	Triclinic, *P* 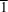
Temperature (K)	100
*a*, *b*, *c* (Å)	9.5599 (4), 11.8056 (5), 17.5322 (7)
α, β, γ (°)	94.808 (2), 104.562 (2), 95.408 (2)
*V* (Å^3^)	1894.70 (14)
*Z*	4
Radiation type	Ag *K*α, λ = 0.56086 Å
μ (mm^−1^)	0.12
Crystal size (mm)	0.32 × 0.28 × 0.25

Data collection	
Diffractometer	Bruker D8 Venture DUO with Photon III C14
Absorption correction	Multi-scan (*SADABS*; Krause *et al.*, 2015 ▸)
*T*_min_, *T*_max_	0.944, 0.970
No. of measured, independent and observed [*I* > 2σ(*I*)] reflections	125520, 15816, 13259
*R*_int_	0.084
(sin θ/λ)_max_ (Å^−1^)	0.794

Refinement	
*R*[*F*^2^ > 2σ(*F*^2^)], *wR*(*F*^2^), *S*	0.063, 0.141, 1.16
No. of reflections	15816
No. of parameters	508
No. of restraints	45
H-atom treatment	H atoms treated by a mixture of independent and constrained refinement
Δρ_max_, Δρ_min_ (e Å^−3^)	0.63, −0.67

Computer programs: *APEX4* and *SAINT* (Bruker, 2022 ▸), *SHELXT2018/2* (Sheldrick, 2015*a* ▸), *SHELXL2019/1* (Sheldrick, 2015*b* ▸), *Mercury* (Macrae *et al.*, 2020 ▸) and *publCIF* (Westrip, 2010 ▸).

## supplementary crystallographic information

### 2,3,5-Triphenyltetrazol-3-ium chloride hemipentahydrate Crystal data

**Table:**

C_19_H_15_N_4_^+^·Cl^−^·2.5H_2_O	*Z* = 4
*M**_r_* = 379.84	*F*(000) = 796
Triclinic, *P*1	*D*_x_ = 1.332 Mg m^−^^3^
*a* = 9.5599 (4) Å	Ag *K*α radiation, λ = 0.56086 Å
*b* = 11.8056 (5) Å	Cell parameters from 9994 reflections
*c* = 17.5322 (7) Å	θ = 2.6–26.3°
α = 94.808 (2)°	µ = 0.12 mm^−^^1^
β = 104.562 (2)°	*T* = 100 K
γ = 95.408 (2)°	Fragment, colourless
*V* = 1894.70 (14) Å^3^	0.32 × 0.28 × 0.25 mm

### 2,3,5-Triphenyltetrazol-3-ium chloride hemipentahydrate Data collection

**Table:**

Bruker D8 Venture DUO with Photon III C14 diffractometer	13259 reflections with *I* > 2σ(*I*)
Radiation source: IµS 3.0 microfocus	*R*_int_ = 0.084
φ and ω scans	θ_max_ = 26.4°, θ_min_ = 2.1°
Absorption correction: multi-scan (SADABS; Krause *et al.*, 2015)	*h* = −15→15
*T*_min_ = 0.944, *T*_max_ = 0.970	*k* = −18→18
125520 measured reflections	*l* = −27→27
15816 independent reflections	

### 2,3,5-Triphenyltetrazol-3-ium chloride hemipentahydrate Refinement

**Table:**

Refinement on *F*^2^	Primary atom site location: dual
Least-squares matrix: full	Hydrogen site location: mixed
*R*[*F*^2^ > 2σ(*F*^2^)] = 0.063	H atoms treated by a mixture of independent and constrained refinement
*wR*(*F*^2^) = 0.141	*w* = 1/[σ^2^(*F*_o_^2^) + (0.0502*P*)^2^ + 1.073*P*] where *P* = (*F*_o_^2^ + 2*F*_c_^2^)/3
*S* = 1.16	(Δ/σ)_max_ = 0.001
15816 reflections	Δρ_max_ = 0.63 e Å^−^^3^
508 parameters	Δρ_min_ = −0.67 e Å^−^^3^
45 restraints	

### 2,3,5-Triphenyltetrazol-3-ium chloride hemipentahydrate Special details

**Table:**

Geometry. All esds (except the esd in the dihedral angle between two l.s. planes) are estimated using the full covariance matrix. The cell esds are taken into account individually in the estimation of esds in distances, angles and torsion angles; correlations between esds in cell parameters are only used when they are defined by crystal symmetry. An approximate (isotropic) treatment of cell esds is used for estimating esds involving l.s. planes.

### 2,3,5-Triphenyltetrazol-3-ium chloride hemipentahydrate Fractional atomic coordinates and isotropic or equivalent isotropic displacement parameters (Å^2^)

**Table:**

	*x*	*y*	*z*	*U*_iso_*/*U*_eq_	
Cl1	0.70828 (4)	0.44966 (3)	0.10654 (2)	0.02287 (8)	
Cl2	0.36722 (5)	0.81273 (4)	0.47669 (2)	0.03148 (10)	
N1	1.06087 (12)	0.41493 (9)	0.30022 (7)	0.01281 (18)	
N2	1.01796 (12)	0.51703 (9)	0.29532 (6)	0.01148 (18)	
N3	0.91361 (12)	0.52776 (9)	0.33271 (6)	0.01127 (18)	
N4	0.88812 (12)	0.43342 (9)	0.36428 (7)	0.01265 (18)	
C1	0.98037 (13)	0.36431 (10)	0.34357 (7)	0.0119 (2)	
C2	0.99419 (14)	0.24860 (10)	0.36518 (7)	0.0127 (2)	
C3	1.11278 (15)	0.19497 (12)	0.35379 (8)	0.0168 (2)	
H3	1.184221	0.234181	0.333244	0.020*	
C4	1.12526 (17)	0.08350 (12)	0.37282 (9)	0.0207 (3)	
H4	1.206066	0.046687	0.365849	0.025*	
C5	1.01958 (19)	0.02616 (12)	0.40200 (9)	0.0226 (3)	
H5	1.027171	−0.050537	0.413628	0.027*	
C6	0.90284 (18)	0.08019 (12)	0.41429 (8)	0.0204 (3)	
H6	0.831886	0.040812	0.435081	0.025*	
C7	0.88968 (15)	0.19187 (11)	0.39619 (8)	0.0154 (2)	
H7	0.810327	0.229196	0.404859	0.019*	
C8	1.08402 (13)	0.60275 (10)	0.25710 (7)	0.0118 (2)	
C9	1.10553 (15)	0.56581 (12)	0.18416 (8)	0.0153 (2)	
H9	1.070334	0.490211	0.159463	0.018*	
C10	1.18016 (16)	0.64285 (13)	0.14837 (8)	0.0186 (2)	
H10	1.197734	0.619924	0.098701	0.022*	
C11	1.22917 (15)	0.75340 (13)	0.18512 (9)	0.0195 (3)	
H11	1.279070	0.806102	0.160079	0.023*	
C12	1.20566 (15)	0.78746 (12)	0.25834 (9)	0.0177 (2)	
H12	1.239657	0.863305	0.282850	0.021*	
C13	1.13283 (14)	0.71156 (11)	0.29605 (8)	0.0145 (2)	
H13	1.117314	0.733548	0.346380	0.017*	
C14	0.83772 (14)	0.62624 (10)	0.33853 (7)	0.0122 (2)	
C15	0.77717 (15)	0.67406 (12)	0.27005 (8)	0.0160 (2)	
H15	0.786806	0.643983	0.219891	0.019*	
C16	0.70196 (16)	0.76752 (13)	0.27755 (9)	0.0210 (3)	
H16	0.661365	0.803899	0.232134	0.025*	
C17	0.68564 (17)	0.80833 (13)	0.35140 (10)	0.0233 (3)	
H17	0.632602	0.871611	0.355801	0.028*	
C18	0.74620 (18)	0.75730 (13)	0.41848 (9)	0.0228 (3)	
H18	0.733652	0.785380	0.468421	0.027*	
C19	0.82497 (16)	0.66550 (12)	0.41304 (8)	0.0176 (2)	
H19	0.868633	0.630747	0.458692	0.021*	
N5	0.34121 (12)	0.77429 (9)	0.78463 (6)	0.01196 (18)	
N6	0.45974 (12)	0.72436 (9)	0.79383 (6)	0.01103 (17)	
N7	0.54655 (11)	0.75476 (9)	0.86647 (6)	0.01081 (17)	
N8	0.48642 (12)	0.82506 (9)	0.90686 (6)	0.01171 (18)	
C20	0.35971 (13)	0.83685 (10)	0.85526 (7)	0.01125 (19)	
C21	0.25809 (14)	0.91367 (11)	0.87263 (7)	0.0126 (2)	
C22	0.16689 (15)	0.95904 (12)	0.81057 (8)	0.0167 (2)	
H22	0.168326	0.937903	0.757308	0.020*	
C23	0.07367 (18)	1.03559 (13)	0.82727 (10)	0.0233 (3)	
H23	0.011239	1.067061	0.785300	0.028*	
C24	0.07175 (18)	1.06608 (14)	0.90525 (10)	0.0257 (3)	
H24	0.007532	1.117997	0.916441	0.031*	
C25	0.16344 (17)	1.02088 (13)	0.96690 (9)	0.0214 (3)	
H25	0.161744	1.042165	1.020099	0.026*	
C26	0.25762 (15)	0.94473 (12)	0.95119 (8)	0.0164 (2)	
H26	0.320861	0.914190	0.993332	0.020*	
C27	0.49263 (13)	0.64728 (11)	0.73433 (7)	0.0121 (2)	
C28	0.52080 (14)	0.53771 (11)	0.75180 (8)	0.0153 (2)	
H28	0.517685	0.512997	0.801599	0.018*	
C29	0.55378 (15)	0.46552 (13)	0.69343 (9)	0.0200 (3)	
H29	0.573212	0.389835	0.703092	0.024*	
C30	0.55841 (16)	0.50361 (15)	0.62116 (9)	0.0237 (3)	
H30	0.582347	0.454021	0.582032	0.028*	
C31	0.52840 (17)	0.61350 (15)	0.60550 (8)	0.0224 (3)	
H31	0.530920	0.638198	0.555610	0.027*	
C32	0.49462 (15)	0.68766 (13)	0.66259 (8)	0.0169 (2)	
H32	0.473763	0.762990	0.652720	0.020*	
C33	0.69328 (13)	0.72747 (11)	0.89345 (7)	0.01130 (19)	
C34	0.79458 (14)	0.76919 (12)	0.85529 (8)	0.0147 (2)	
H34	0.767638	0.812168	0.811542	0.018*	
C35	0.93677 (14)	0.74586 (13)	0.88334 (8)	0.0176 (2)	
H35	1.008994	0.772656	0.858364	0.021*	
C36	0.97440 (15)	0.68333 (13)	0.94797 (8)	0.0184 (2)	
H36	1.072249	0.668184	0.966914	0.022*	
C37	0.86978 (15)	0.64294 (13)	0.98493 (8)	0.0181 (2)	
H37	0.896309	0.600303	1.028887	0.022*	
C38	0.72645 (14)	0.66494 (12)	0.95758 (8)	0.0149 (2)	
H38	0.653676	0.637922	0.982119	0.018*	
O1	0.83271 (13)	0.41876 (10)	0.53365 (6)	0.0209 (2)	
H1W	0.780 (2)	0.3554 (14)	0.5308 (14)	0.031*	
H2W	0.828 (3)	0.430 (2)	0.4856 (9)	0.031*	
O2	0.53149 (13)	0.99355 (10)	0.61966 (6)	0.0212 (2)	
H3W	0.484 (2)	0.9458 (17)	0.5819 (11)	0.032*	
H4W	0.558 (3)	1.0481 (16)	0.5968 (13)	0.032*	
O3	0.57974 (15)	0.96751 (10)	0.78259 (7)	0.0254 (2)	
H5W	0.564 (3)	0.980 (2)	0.7343 (9)	0.038*	
H6W	0.556 (3)	1.0222 (17)	0.8094 (13)	0.038*	
O4	0.46846 (15)	0.62378 (11)	0.07228 (8)	0.0277 (2)	
H7W	0.420 (3)	0.605 (2)	0.0242 (9)	0.041*	
H8W	0.533 (2)	0.5782 (19)	0.0806 (15)	0.041*	
O5	0.49505 (13)	1.14221 (10)	0.89204 (7)	0.0219 (2)	
H9W	0.502 (3)	1.2147 (12)	0.9044 (14)	0.033*	
H10W	0.435 (2)	1.1154 (19)	0.9169 (13)	0.033*	

### 2,3,5-Triphenyltetrazol-3-ium chloride hemipentahydrate Atomic displacement parameters (Å^2^)

**Table:**

	*U*^11^	*U*^22^	*U*^33^	*U*^12^	*U*^13^	*U*^23^
Cl1	0.02495 (17)	0.01712 (15)	0.02952 (18)	−0.00068 (12)	0.01420 (14)	0.00123 (12)
Cl2	0.0403 (2)	0.0297 (2)	0.01840 (16)	−0.01646 (17)	0.00415 (15)	0.00281 (13)
N1	0.0144 (5)	0.0092 (4)	0.0156 (5)	0.0019 (3)	0.0053 (4)	0.0011 (3)
N2	0.0119 (4)	0.0099 (4)	0.0132 (4)	0.0011 (3)	0.0047 (3)	0.0006 (3)
N3	0.0112 (4)	0.0095 (4)	0.0137 (4)	0.0012 (3)	0.0044 (3)	0.0010 (3)
N4	0.0132 (5)	0.0093 (4)	0.0157 (5)	0.0009 (3)	0.0041 (4)	0.0019 (3)
C1	0.0115 (5)	0.0101 (5)	0.0136 (5)	0.0010 (4)	0.0031 (4)	0.0003 (4)
C2	0.0143 (5)	0.0089 (5)	0.0141 (5)	0.0014 (4)	0.0024 (4)	0.0008 (4)
C3	0.0162 (6)	0.0147 (5)	0.0191 (6)	0.0041 (4)	0.0034 (4)	0.0001 (4)
C4	0.0234 (7)	0.0164 (6)	0.0212 (6)	0.0087 (5)	0.0017 (5)	0.0005 (5)
C5	0.0344 (8)	0.0123 (6)	0.0197 (6)	0.0065 (5)	0.0023 (6)	0.0040 (5)
C6	0.0301 (7)	0.0132 (5)	0.0172 (6)	−0.0009 (5)	0.0055 (5)	0.0033 (4)
C7	0.0182 (6)	0.0128 (5)	0.0148 (5)	0.0005 (4)	0.0039 (4)	0.0014 (4)
C8	0.0110 (5)	0.0104 (5)	0.0141 (5)	−0.0001 (4)	0.0035 (4)	0.0027 (4)
C9	0.0155 (6)	0.0155 (5)	0.0151 (5)	0.0002 (4)	0.0051 (4)	0.0013 (4)
C10	0.0174 (6)	0.0224 (6)	0.0176 (6)	0.0010 (5)	0.0071 (5)	0.0057 (5)
C11	0.0141 (6)	0.0202 (6)	0.0249 (6)	−0.0008 (5)	0.0053 (5)	0.0094 (5)
C12	0.0153 (6)	0.0126 (5)	0.0240 (6)	−0.0014 (4)	0.0036 (5)	0.0031 (5)
C13	0.0131 (5)	0.0121 (5)	0.0171 (5)	0.0004 (4)	0.0025 (4)	0.0008 (4)
C14	0.0113 (5)	0.0095 (5)	0.0155 (5)	0.0017 (4)	0.0028 (4)	0.0001 (4)
C15	0.0141 (5)	0.0162 (5)	0.0168 (5)	0.0031 (4)	0.0017 (4)	0.0026 (4)
C16	0.0168 (6)	0.0173 (6)	0.0272 (7)	0.0054 (5)	0.0001 (5)	0.0053 (5)
C17	0.0188 (6)	0.0154 (6)	0.0339 (8)	0.0072 (5)	0.0036 (6)	−0.0023 (5)
C18	0.0247 (7)	0.0195 (6)	0.0238 (7)	0.0064 (5)	0.0067 (5)	−0.0054 (5)
C19	0.0198 (6)	0.0168 (6)	0.0162 (5)	0.0057 (5)	0.0041 (5)	−0.0012 (4)
N5	0.0105 (4)	0.0126 (4)	0.0125 (4)	0.0020 (3)	0.0026 (3)	0.0003 (3)
N6	0.0102 (4)	0.0119 (4)	0.0098 (4)	0.0003 (3)	0.0012 (3)	−0.0002 (3)
N7	0.0093 (4)	0.0126 (4)	0.0097 (4)	−0.0001 (3)	0.0022 (3)	−0.0008 (3)
N8	0.0100 (4)	0.0132 (4)	0.0118 (4)	0.0004 (3)	0.0037 (3)	−0.0005 (3)
C20	0.0104 (5)	0.0112 (5)	0.0121 (5)	−0.0004 (4)	0.0037 (4)	0.0006 (4)
C21	0.0112 (5)	0.0115 (5)	0.0146 (5)	0.0005 (4)	0.0036 (4)	−0.0014 (4)
C22	0.0182 (6)	0.0145 (5)	0.0170 (5)	0.0042 (4)	0.0037 (5)	0.0005 (4)
C23	0.0243 (7)	0.0189 (6)	0.0261 (7)	0.0102 (5)	0.0037 (6)	0.0000 (5)
C24	0.0239 (7)	0.0206 (7)	0.0320 (8)	0.0076 (5)	0.0079 (6)	−0.0086 (6)
C25	0.0191 (6)	0.0226 (7)	0.0216 (6)	0.0007 (5)	0.0078 (5)	−0.0085 (5)
C26	0.0138 (5)	0.0192 (6)	0.0147 (5)	−0.0001 (4)	0.0034 (4)	−0.0031 (4)
C27	0.0099 (5)	0.0141 (5)	0.0115 (5)	0.0005 (4)	0.0028 (4)	−0.0028 (4)
C28	0.0120 (5)	0.0148 (5)	0.0178 (5)	0.0012 (4)	0.0026 (4)	−0.0010 (4)
C29	0.0137 (6)	0.0188 (6)	0.0245 (6)	0.0038 (5)	0.0020 (5)	−0.0070 (5)
C30	0.0152 (6)	0.0338 (8)	0.0194 (6)	0.0061 (5)	0.0030 (5)	−0.0111 (6)
C31	0.0188 (6)	0.0358 (8)	0.0124 (5)	0.0046 (6)	0.0048 (5)	−0.0026 (5)
C32	0.0150 (6)	0.0224 (6)	0.0124 (5)	0.0022 (5)	0.0029 (4)	0.0004 (4)
C33	0.0086 (5)	0.0132 (5)	0.0114 (5)	0.0003 (4)	0.0022 (4)	−0.0005 (4)
C34	0.0129 (5)	0.0172 (5)	0.0133 (5)	−0.0023 (4)	0.0041 (4)	0.0002 (4)
C35	0.0106 (5)	0.0225 (6)	0.0188 (6)	−0.0032 (4)	0.0057 (4)	−0.0029 (5)
C36	0.0105 (5)	0.0223 (6)	0.0201 (6)	0.0021 (5)	0.0019 (4)	−0.0032 (5)
C37	0.0146 (6)	0.0211 (6)	0.0169 (6)	0.0031 (5)	0.0003 (4)	0.0034 (5)
C38	0.0124 (5)	0.0176 (6)	0.0148 (5)	0.0010 (4)	0.0037 (4)	0.0033 (4)
O1	0.0232 (5)	0.0212 (5)	0.0167 (4)	−0.0017 (4)	0.0049 (4)	0.0001 (4)
O2	0.0286 (6)	0.0174 (5)	0.0175 (5)	0.0028 (4)	0.0063 (4)	0.0006 (4)
O3	0.0351 (6)	0.0179 (5)	0.0252 (5)	0.0014 (4)	0.0106 (5)	0.0064 (4)
O4	0.0305 (6)	0.0227 (5)	0.0326 (6)	0.0015 (5)	0.0165 (5)	−0.0037 (5)
O5	0.0256 (5)	0.0187 (5)	0.0214 (5)	−0.0009 (4)	0.0070 (4)	0.0037 (4)

### 2,3,5-Triphenyltetrazol-3-ium chloride hemipentahydrate Geometric parameters (Å, º)

**Table:**

N1—N2	1.3120 (15)	N7—C33	1.4395 (16)
N1—C1	1.3455 (16)	N8—C20	1.3442 (16)
N2—N3	1.3341 (14)	C20—C21	1.4586 (17)
N2—C8	1.4415 (16)	C21—C22	1.3927 (19)
N3—N4	1.3137 (15)	C21—C26	1.3965 (18)
N3—C14	1.4370 (16)	C22—C23	1.391 (2)
N4—C1	1.3500 (16)	C22—H22	0.9500
C1—C2	1.4569 (17)	C23—C24	1.389 (2)
C2—C7	1.3966 (18)	C23—H23	0.9500
C2—C3	1.3979 (18)	C24—C25	1.389 (2)
C3—C4	1.393 (2)	C24—H24	0.9500
C3—H3	0.9500	C25—C26	1.389 (2)
C4—C5	1.389 (2)	C25—H25	0.9500
C4—H4	0.9500	C26—H26	0.9500
C5—C6	1.390 (2)	C27—C32	1.3858 (18)
C5—H5	0.9500	C27—C28	1.3893 (19)
C6—C7	1.3914 (19)	C28—C29	1.3927 (19)
C6—H6	0.9500	C28—H28	0.9500
C7—H7	0.9500	C29—C30	1.389 (2)
C8—C13	1.3835 (17)	C29—H29	0.9500
C8—C9	1.3870 (18)	C30—C31	1.390 (2)
C9—C10	1.3886 (19)	C30—H30	0.9500
C9—H9	0.9500	C31—C32	1.3934 (19)
C10—C11	1.389 (2)	C31—H31	0.9500
C10—H10	0.9500	C32—H32	0.9500
C11—C12	1.391 (2)	C33—C38	1.3834 (18)
C11—H11	0.9500	C33—C34	1.3854 (17)
C12—C13	1.3922 (19)	C34—C35	1.3866 (19)
C12—H12	0.9500	C34—H34	0.9500
C13—H13	0.9500	C35—C36	1.394 (2)
C14—C15	1.3859 (18)	C35—H35	0.9500
C14—C19	1.3877 (18)	C36—C37	1.391 (2)
C15—C16	1.387 (2)	C36—H36	0.9500
C15—H15	0.9500	C37—C38	1.3902 (19)
C16—C17	1.394 (2)	C37—H37	0.9500
C16—H16	0.9500	C38—H38	0.9500
C17—C18	1.387 (2)	O1—H1W	0.851 (14)
C17—H17	0.9500	O1—H2W	0.853 (14)
C18—C19	1.387 (2)	O2—H3W	0.836 (14)
C18—H18	0.9500	O2—H4W	0.838 (14)
C19—H19	0.9500	O3—H5W	0.849 (14)
N5—N6	1.3066 (15)	O3—H6W	0.849 (14)
N5—C20	1.3498 (16)	O4—H7W	0.852 (14)
N6—N7	1.3324 (14)	O4—H8W	0.849 (14)
N6—C27	1.4404 (16)	O5—H9W	0.857 (14)
N7—N8	1.3103 (14)	O5—H10W	0.854 (14)
			
N2—N1—C1	103.93 (10)	N7—N6—C27	124.54 (10)
N1—N2—N3	110.20 (10)	N8—N7—N6	110.23 (10)
N1—N2—C8	122.11 (10)	N8—N7—C33	124.23 (10)
N3—N2—C8	127.63 (10)	N6—N7—C33	125.03 (10)
N4—N3—N2	109.93 (10)	N7—N8—C20	103.56 (10)
N4—N3—C14	123.73 (10)	N8—C20—N5	112.38 (11)
N2—N3—C14	126.34 (10)	N8—C20—C21	123.45 (11)
N3—N4—C1	103.88 (10)	N5—C20—C21	124.09 (11)
N1—C1—N4	112.03 (11)	C22—C21—C26	120.79 (12)
N1—C1—C2	122.91 (11)	C22—C21—C20	119.33 (11)
N4—C1—C2	125.05 (11)	C26—C21—C20	119.83 (12)
C7—C2—C3	120.57 (12)	C23—C22—C21	119.41 (13)
C7—C2—C1	120.39 (11)	C23—C22—H22	120.3
C3—C2—C1	119.04 (12)	C21—C22—H22	120.3
C4—C3—C2	119.40 (13)	C24—C23—C22	120.09 (15)
C4—C3—H3	120.3	C24—C23—H23	120.0
C2—C3—H3	120.3	C22—C23—H23	120.0
C5—C4—C3	120.02 (13)	C25—C24—C23	120.21 (14)
C5—C4—H4	120.0	C25—C24—H24	119.9
C3—C4—H4	120.0	C23—C24—H24	119.9
C4—C5—C6	120.46 (13)	C24—C25—C26	120.37 (13)
C4—C5—H5	119.8	C24—C25—H25	119.8
C6—C5—H5	119.8	C26—C25—H25	119.8
C5—C6—C7	120.12 (14)	C25—C26—C21	119.12 (13)
C5—C6—H6	119.9	C25—C26—H26	120.4
C7—C6—H6	119.9	C21—C26—H26	120.4
C6—C7—C2	119.40 (13)	C32—C27—C28	123.81 (12)
C6—C7—H7	120.3	C32—C27—N6	117.50 (12)
C2—C7—H7	120.3	C28—C27—N6	118.68 (11)
C13—C8—C9	123.75 (12)	C27—C28—C29	117.38 (13)
C13—C8—N2	120.11 (11)	C27—C28—H28	121.3
C9—C8—N2	115.91 (11)	C29—C28—H28	121.3
C8—C9—C10	117.88 (12)	C30—C29—C28	120.35 (14)
C8—C9—H9	121.1	C30—C29—H29	119.8
C10—C9—H9	121.1	C28—C29—H29	119.8
C9—C10—C11	120.09 (13)	C29—C30—C31	120.68 (13)
C9—C10—H10	120.0	C29—C30—H30	119.7
C11—C10—H10	120.0	C31—C30—H30	119.7
C10—C11—C12	120.44 (13)	C30—C31—C32	120.33 (14)
C10—C11—H11	119.8	C30—C31—H31	119.8
C12—C11—H11	119.8	C32—C31—H31	119.8
C11—C12—C13	120.70 (13)	C27—C32—C31	117.43 (14)
C11—C12—H12	119.7	C27—C32—H32	121.3
C13—C12—H12	119.7	C31—C32—H32	121.3
C8—C13—C12	117.14 (12)	C38—C33—C34	123.74 (12)
C8—C13—H13	121.4	C38—C33—N7	118.40 (11)
C12—C13—H13	121.4	C34—C33—N7	117.82 (11)
C15—C14—C19	123.67 (12)	C33—C34—C35	117.50 (12)
C15—C14—N3	118.95 (11)	C33—C34—H34	121.3
C19—C14—N3	117.34 (11)	C35—C34—H34	121.3
C14—C15—C16	117.46 (13)	C34—C35—C36	120.40 (12)
C14—C15—H15	121.3	C34—C35—H35	119.8
C16—C15—H15	121.3	C36—C35—H35	119.8
C15—C16—C17	120.35 (14)	C37—C36—C35	120.51 (13)
C15—C16—H16	119.8	C37—C36—H36	119.7
C17—C16—H16	119.8	C35—C36—H36	119.7
C18—C17—C16	120.54 (13)	C38—C37—C36	120.04 (13)
C18—C17—H17	119.7	C38—C37—H37	120.0
C16—C17—H17	119.7	C36—C37—H37	120.0
C19—C18—C17	120.35 (14)	C33—C38—C37	117.80 (12)
C19—C18—H18	119.8	C33—C38—H38	121.1
C17—C18—H18	119.8	C37—C38—H38	121.1
C18—C19—C14	117.61 (13)	H1W—O1—H2W	105 (2)
C18—C19—H19	121.2	H3W—O2—H4W	103 (2)
C14—C19—H19	121.2	H5W—O3—H6W	110 (2)
N6—N5—C20	103.54 (10)	H7W—O4—H8W	105 (2)
N5—N6—N7	110.29 (10)	H9W—O5—H10W	103 (2)
N5—N6—C27	125.17 (10)		
			
C1—N1—N2—N3	−1.18 (13)	C20—N5—N6—N7	0.02 (13)
C1—N1—N2—C8	176.26 (11)	C20—N5—N6—C27	179.94 (11)
N1—N2—N3—N4	1.26 (14)	N5—N6—N7—N8	0.34 (14)
C8—N2—N3—N4	−175.99 (11)	C27—N6—N7—N8	−179.58 (11)
N1—N2—N3—C14	−178.51 (11)	N5—N6—N7—C33	−171.74 (11)
C8—N2—N3—C14	4.2 (2)	C27—N6—N7—C33	8.34 (18)
N2—N3—N4—C1	−0.74 (13)	N6—N7—N8—C20	−0.53 (13)
C14—N3—N4—C1	179.04 (11)	C33—N7—N8—C20	171.62 (11)
N2—N1—C1—N4	0.73 (14)	N7—N8—C20—N5	0.56 (14)
N2—N1—C1—C2	−178.88 (11)	N7—N8—C20—C21	−176.25 (11)
N3—N4—C1—N1	0.00 (14)	N6—N5—C20—N8	−0.37 (14)
N3—N4—C1—C2	179.60 (12)	N6—N5—C20—C21	176.42 (11)
N1—C1—C2—C7	−167.19 (12)	N8—C20—C21—C22	152.30 (13)
N4—C1—C2—C7	13.25 (19)	N5—C20—C21—C22	−24.14 (19)
N1—C1—C2—C3	12.26 (19)	N8—C20—C21—C26	−24.95 (19)
N4—C1—C2—C3	−167.30 (13)	N5—C20—C21—C26	158.61 (12)
C7—C2—C3—C4	0.7 (2)	C26—C21—C22—C23	−0.4 (2)
C1—C2—C3—C4	−178.73 (12)	C20—C21—C22—C23	−177.66 (13)
C2—C3—C4—C5	0.7 (2)	C21—C22—C23—C24	−0.1 (2)
C3—C4—C5—C6	−1.6 (2)	C22—C23—C24—C25	0.4 (3)
C4—C5—C6—C7	1.0 (2)	C23—C24—C25—C26	−0.1 (2)
C5—C6—C7—C2	0.4 (2)	C24—C25—C26—C21	−0.4 (2)
C3—C2—C7—C6	−1.3 (2)	C22—C21—C26—C25	0.7 (2)
C1—C2—C7—C6	178.16 (12)	C20—C21—C26—C25	177.93 (12)
N1—N2—C8—C13	−128.71 (13)	N5—N6—C27—C32	57.53 (17)
N3—N2—C8—C13	48.25 (18)	N7—N6—C27—C32	−122.57 (13)
N1—N2—C8—C9	45.93 (17)	N5—N6—C27—C28	−122.66 (14)
N3—N2—C8—C9	−137.11 (13)	N7—N6—C27—C28	57.24 (17)
C13—C8—C9—C10	−0.1 (2)	C32—C27—C28—C29	0.5 (2)
N2—C8—C9—C10	−174.54 (12)	N6—C27—C28—C29	−179.33 (12)
C8—C9—C10—C11	−0.8 (2)	C27—C28—C29—C30	0.3 (2)
C9—C10—C11—C12	0.8 (2)	C28—C29—C30—C31	−0.9 (2)
C10—C11—C12—C13	0.0 (2)	C29—C30—C31—C32	0.7 (2)
C9—C8—C13—C12	0.9 (2)	C28—C27—C32—C31	−0.6 (2)
N2—C8—C13—C12	175.12 (12)	N6—C27—C32—C31	179.15 (12)
C11—C12—C13—C8	−0.9 (2)	C30—C31—C32—C27	0.1 (2)
N4—N3—C14—C15	−129.44 (13)	N8—N7—C33—C38	68.04 (16)
N2—N3—C14—C15	50.30 (18)	N6—N7—C33—C38	−120.96 (13)
N4—N3—C14—C19	48.26 (17)	N8—N7—C33—C34	−109.63 (14)
N2—N3—C14—C19	−132.00 (13)	N6—N7—C33—C34	61.37 (17)
C19—C14—C15—C16	1.1 (2)	C38—C33—C34—C35	0.3 (2)
N3—C14—C15—C16	178.63 (12)	N7—C33—C34—C35	177.85 (11)
C14—C15—C16—C17	−1.8 (2)	C33—C34—C35—C36	−0.5 (2)
C15—C16—C17—C18	1.0 (2)	C34—C35—C36—C37	0.4 (2)
C16—C17—C18—C19	0.6 (2)	C35—C36—C37—C38	−0.1 (2)
C17—C18—C19—C14	−1.3 (2)	C34—C33—C38—C37	−0.1 (2)
C15—C14—C19—C18	0.4 (2)	N7—C33—C38—C37	−177.58 (12)
N3—C14—C19—C18	−177.15 (13)	C36—C37—C38—C33	−0.1 (2)

### 2,3,5-Triphenyltetrazol-3-ium chloride hemipentahydrate Hydrogen-bond geometry (Å, º)

*Cg*6 is the centroid of the C21–C26 ring.

**Table:**

*D*—H···*A*	*D*—H	H···*A*	*D*···*A*	*D*—H···*A*
O1—H1*W*···Cl2^i^	0.85 (1)	2.30 (1)	3.1483 (12)	178 (2)
O1—H2*W*···N4	0.85 (1)	2.34 (2)	3.1605 (15)	160 (2)
O2—H3*W*···Cl2	0.84 (1)	2.30 (1)	3.1302 (12)	176 (2)
O2—H4*W*···Cl2^ii^	0.84 (1)	2.34 (1)	3.1740 (12)	175 (2)
O3—H5*W*···O2	0.85 (1)	1.98 (1)	2.8246 (16)	175 (2)
O3—H6*W*···O5	0.85 (1)	2.16 (2)	3.0040 (17)	171 (2)
O4—H7*W*···Cl1^iii^	0.85 (1)	2.32 (2)	3.1687 (14)	179 (3)
O4—H8*W*···Cl1	0.85 (1)	2.35 (2)	3.2008 (14)	178 (2)
O5—H9*W*···O4^ii^	0.86 (1)	1.90 (1)	2.7503 (17)	175 (2)
O5—H10*W*···*Cg*6	0.85 (1)	2.76 (2)	3.4646 (14)	141 (2)
C4—H4···O2^iv^	0.95	2.55	3.461 (2)	160
C11—H11···O5^v^	0.95	2.59	3.429 (2)	147
C15—H15···Cl1	0.95	2.82	3.6219 (14)	143
C16—H16···O5^ii^	0.95	2.48	3.4084 (19)	165
C17—H17···O2^ii^	0.95	2.42	3.359 (2)	168
C26—H26···O5^vi^	0.95	2.50	3.4440 (19)	173

Symmetry codes: (i) −*x*+1, −*y*+1, −*z*+1; (ii) −*x*+1, −*y*+2, −*z*+1; (iii) −*x*+1, −*y*+1, −*z*; (iv) −*x*+2, −*y*+1, −*z*+1; (v) −*x*+2, −*y*+2, −*z*+1; (vi) −*x*+1, −*y*+2, −*z*+2.
